# Prodigiosin - A Multifaceted *Escherichia coli* Antimicrobial Agent

**DOI:** 10.1371/journal.pone.0162412

**Published:** 2016-09-09

**Authors:** Tjaša Danevčič, Maja Borić Vezjak, Maša Zorec, David Stopar

**Affiliations:** 1 Laboratory of Microbiology, Department of Food Science and Technology, Biotechnical Faculty, University of Ljubljana, Ljubljana, Slovenia; 2 Laboratory of Microbiology and Microbial Biotechnology, Department of Animal Science, Biotechnical Faculty, University of Ljubljana, Ljubljana, Slovenia; University of Oklahoma, UNITED STATES

## Abstract

Despite a considerable interest in prodigiosin, the mechanism of its antibacterial activity is still poorly understood. In this work, *Escherichia coli* cells were treated with prodigiosin to determine its antimicrobial effect on bacterial physiology. The effect of prodigiosin was concentration dependent. In prodigiosin treated cells above MIC value no significant DNA damage or cytoplasmic membrane disintegration was observed. The outer membrane, however, becomes leaky. Cells had severely decreased respiration activity. In prodigiosin treated cells protein and RNA synthesis were inhibited, cells were elongated but could not divide. Pre-treatment with prodigiosin improved *E*. *coli* survival rate in media containing ampicillin, kanamycin and erythromycin but not phleomycin. The results suggest that prodigiosin acts as a bacteriostatic agent in *E*. *coli* cells. If prodigiosin was diluted, cells resumed growth. The results indicate that prodigiosin has distinct mode of antibacterial action in different bacteria.

## Introduction

Bacteria living in natural environment face different stress factors (i.e. temperature, salinity, water activity, starvation, competition). Microorganisms exposed to antimicrobial stressors have evolved a variety of specific adaptive and protective mechanisms, which include changes in cell membrane, DNA, RNA and protein synthesis, gene expression, biofilm formation, and production of small bioactive molecules [1,2]. In particular small bioactive compounds can shape metabolism and enhance survival of bacterial community in the environment [1].

The red pigment prodigiosin is produced as a secondary metabolite by many bacterial species [3,4]. It has an antibacterial, antiprotozoal, anti-tumor and anti-inflammatory activity [3,5–8]. Despite decades of research, the mechanism underlying its antibacterial activity remains poorly explained. It has been demonstrated that prodigiosin inhibits growth of a wide spectrum of Gram positive bacteria including *Bacillus subtilis* and *Staphylococcus aureus*, as well as Gram negative *Escherichia coli*, *Salmonella enterica* and *Erwinia carotovora* [3,9–15]. For a producer to survive it should be self-resistant to prodigiosin. It has been shown that in prodigiosin producing *Streptomyces griseoviridis* species *rphA1* and *rphA2* ABC-transporter genes are important for self-resistance [16]. The bacteria that do not have such homologues are prodigiosin sensitive. Prodigiosin as an antibacterial agent has high potential in biotechnological and medical applications [4–11], as well as in microbial ecology due to its ability to modulate bacterial ecophysiology [12,17–19]. It was shown that prodigiosin impregnated to cellulose matrix effectively removes *E*. *coli* and *B*. *cereus* from contaminated water [20]. Recently the induction of autolysins in *Bacillus subtilis* and other *Bacillus* species has been demonstrated as a potent antibacterial mechanism [15]. Different studies [11,13] reported inhibitory effect of prodigiosin on *E*. *coli*. The MIC value of prodigiosin for *E*. *coli* MG1655 was determined to be 103.4 ± 6.3 mg L^-1^ [15], which is moderate compared to some other known *E*. *coli* antimicrobial agents [21]. On the other hand, several studies showed no effect of prodigiosin on *E*. *coli* cells [22,23]. Because the mechanism of prodigiosin´s action on *E*. *coli* is not known, the conflicting data of prodigiosin antibacterial action are difficult to reconcile. As several ecopysiological roles of prodigiosin have been proposed for bacteria such as air dispersal of bacteria [24], metabolic sink for NAD(P)H or proline [25], storage of light energy [17], anion exchange [18], energy spilling function [26] and UV protection [19], it is possible that antimicrobial activity is not the result of prodigiosin aiming at a single cell target, but in turn may have a pleiotropic effect on *E*. *coli* physiology. Many antimicrobial agents are indeed known to have multiple effects on microorganisms [27].

In this study, the effect of prodigiosin on *E*. *coli* physiological behaviour was studied. Different modes of prodigiosin antibacterial activity were tested, including DNA cleavage, induction of SOS response, the effect on cell membrane integrity, metabolic activity, as well as survival of prodigiosin pre-treated cells in media containing different antibiotics. The results indicate a multifaceted physiological response to prodigiosin in *E*. *coli*, very different from antimicrobial effect of prodigiosin described in a model bacterium *Bacillus subtilis*.

## Materials and Methods

### Prodigiosin extract

Prodigiosin was extracted from *Vibrio* sp. DSM 14379, its purity and concentration were determined as described previously by Danevčič *et al*. [15]. The purity of prodigiosin in the extract was checked by HPLC in the wavelength range from 400 to 600 nm. The extract contained more than 98% of α and β prodigiosin isomers. Prodigiosin concentration in stock solution was 2.93 g L^-1^ in ethanol.

### Bacterial growth and treatment with different prodigiosin concentrations

Bacterial strain *E*. *coli* MG1655 was grown in liquid LB medium at 37°C and 200 rpm. Overnight cultures were diluted 100-fold in 20 mL of LB medium and incubated until the culture reached optical density (OD_650_) between 0.4 and 0.5, *E*. *coli* cells were then treated with 10, 60 or 120 mg L^-1^ of prodigiosin. As a control, an equivalent amount of sterile 96% (V/V) ethanol was added to the culture to the maximal final concentration of 4.44% (V/V).

To test whether *E*. *coli* cells can develop resistance to prodigiosin, cells were treated with 120 mg L^-1^ of prodigiosin in LB medium at 37°C and 200 rpm for 21.5 h and then diluted 100-fold in 20 mL of fresh LB medium. When cells resumed growth and reached OD_650_ 0.4, they were treated again with 120 mg L^-1^ of prodigiosin and incubated further at the same growth conditions. This procedure was repeated 3 times. In all consecutive treatments, CFU counts were determined at the time of treatment and 21.5 h afterwards. Then the Malthusian fitness of the strain was calculated as a natural logarithm of the ratio between the final and initial CFU counts [28].

### Cell morphology, viability, and membrane integrity

*E*. *coli* cells were treated with 120 mg L^-1^ of prodigiosin or 4.44% (V/V) of ethanol (control) in the middle of the exponential phase at OD_650_ between 0.4 and 0.5. Cell morphology was inspected under the inverted epifluorescence microscope Axio Observer Z1 (Carl Zeiss, Germany) at 0.5, 1, 3, 5, and 21.5 hours after addition of prodigiosin or ethanol. The cell length was measured using AxioVision program version 4.8. Additionally, treated *E*. *coli* cells were stained with Live/Dead BacLight bacterial viability assay (Invitrogen, USA) according to the manufacturer instructions at 0, 1, and 5 hours after prodigiosin or ethanol addition. Fluorescence was observed with Axio Observer Z1 using appropriate filter sets for green fluorescent dye SYTO 9 and red fluorescent dye propidium iodide.

### Modified comet assay

*E*. *coli* cells were grown in liquid LB medium as described above. At OD_650_ around 0.5 cells were either treated with 120 mg L^-1^ of prodigiosin, 4.44% (V/V) of ethanol (a negative control) or 100 mg L^-1^ of ampicillin (a positive control). Cells were then incubated for 1 h at 37°C and 200 rpm. For the analysis minigels were prepared and observed as described previously by Danevčič *et al*. [15].

### Nitrocefin hydrolysis assay

The outer membrane leakage was determined as nitrocefin hydrolysis according to the modified method by Mensa *et al*. [29]. Briefly, *E*. *coli* cells were grown overnight in liquid LB medium, diluted 100-fold in fresh LB medium and grown at 37°C and 200 rpm up to OD_650_ 0.6. Cells were harvested by centrifugation and concentrated 5-fold in phosphate saline buffer (137 mM NaCl, 2.7 mM KCl, 10 mM Na_2_HPO_4_, 2 mM KH_2_PO_4_, pH value 7.4). Prodigiosin was added to the cells in the final concentration of 120 mg L^-1^. As a control 4.44% (V/V) ethanol was used. The mixture was incubated at 37°C and 200 rpm for either 1 or 5 hours. At the end of the incubation, nitrocefin (10 mg mL^-1^ in dimethyl sulphoxide, Calbiochem, USA) was added to the mixture to obtain the final concentration 50 μg mL^-1^. The kinetics of nitrocefin hydrolysis was measured spectrophotometrically for 10 min at 486 nm and room temperature in 30 s intervals. The rate of nitrocefin hydrolysis was calculated from the slope of the linear part of the kinetic curves. Nitrocefin hydrolysis provides a measure of β—lactamase activity expressed as the amount of nitrocefin hydrolysed per minute per mg cell protein or U per mg cell protein. To determine the protein content cell extracts were prepared as described by Danevčič and Stopar [30]. Protein content in the cell extracts was determined using Bradford reagent (Sigma, USA) according to the manufacturer instructions.

### Prodigiosin incorporation into bacterial cells

The ability to incorporate prodigiosin into bacterial cells was studied in viable and autoclaved *E*. *coli* MG1655 cells. Bacterial cells were cultured in liquid LB medium as described above. When reaching OD_650_ between 0.4 and 0.5, cells were harvested with centrifugation and washed with 0.9% (w/V) NaCl. Then, one half of the suspension was autoclaved at 121°C and 1.3 bar for 15 min, while the rest was incubated further at room temperature. Next, autoclaved cells were washed again. Both cell suspensions were treated with 120 mg L^-1^ of prodigiosin and incubated at 37°C and 200 rpm. After 5 h of incubation cells were harvested and pigment was extracted and quantified as described previously Borić *et al*. [19]. As a control, cells were treated with 4.44% (V/V) of ethanol.

### Induction of the SOS response and DNA cleavage

*E*. *coli* MG1655 cells transformed with pSC101 bearing *sulA* promoter fused to *gfp* gene [31] were treated with various prodigiosin concentrations (10, 60 and 120 mg L^-1^) or 4.44% (V/V) ethanol as a control. After 30 min of incubation at 37°C and 200 rpm, bacterial cells were inspected under the inverted microscope Axio Observer Z1 using filter set for GFP. The control for the SOS response induction was 30 s exposure of bacterial culture to UVC radiation. Chromosomal DNA was extracted from untreated, as well as from prodigiosin and ethanol treated *E*. *coli* MG1655 cells, using GenElute Bacterial Genomic DNA Kit (Sigma-Aldrich, USA) according to the manufacturer instructions. Isolated chromosomal DNA from untreated *E*. *coli* cells was treated with 60 or 120 mg L^-1^ of prodigiosin for 30 min and then loaded on 0.8% (w/V) agarose gel to check for DNA degradation. Gel electrophoresis was run at 75 V for 1 h. DNA was visualized by ethidium bromide using UV light.

### CO_2_ production

*E*. *coli* MG1655 cells were grown in LB medium at 37°C and 200 rpm overnight. Overnight cultures were diluted 100-fold in 25 mL of fresh LB medium and closed in an air tight 125-mL flask. The amount of CO_2_ produced was monitored on gas chromatograph HP5890A (Hewlett Packard, USA) in regular time intervals as described before Danevčič and Stopar [30] and Ivančič *et al*. [32]. Cells were treated with 120 mg L^-1^ of prodigiosin or 4.44% (V/V) ethanol as a control after 2.5 h of growth. Respiration activity was expressed as the volume of CO_2_ produced.

### Protein and total RNA content

*E*. *coli* cells were grown in liquid LB medium as described above and treated with 120 mg L^-1^ of prodigiosin or 4.44% (V/V) ethanol as a control in the middle of the exponential phase at OD_650_ between 0.4 and 0.5. Total protein content was measured in bacterial extracts prepared from untreated, ethanol, or prodigiosin treated cells according to Danevčič and Stopar [30]. Protein content in cell extracts was measured using Bradford reagent (Sigma-Aldrich, USA) according to the manufacturer instructions. Total RNA from untreated, ethanol, or prodigiosin treated cells was extracted with RNeasy Mini kit (Qiagen, USA) according to the manufacturer instructions and quantified with NanoDrop 1000 (Thermo Scientific, USA).

### Antibiotic treatment survival with prodigiosin pre-treatment

Survival of *E*. *coli* MG1655 cells after treatment with 100 mg L^-1^ ampicillin, 100 mg L^-1^ erythromycin, 100 mg L^-1^ kanamycin and 5 mg L^-1^ phleomycin (Sigma Aldrich, USA) was assessed according to Dӧrr *et al*. [33]. Overnight cultures were diluted 100-fold in 100 mL of fresh LB medium and incubated at 37°C and 200 rpm. After reaching OD_650_ between 0.4 and 0.5, cultures were treated with 120 mg L^-1^ of prodigiosin, or 4.44% (V/V) ethanol, or sterile deionized water and incubated at 37°C and 200 rpm for additional 2 h. Aliquots of 5 mL were then transferred into separate tubes and antibiotics were added. Sterile deionized water was used as a control. Tubes were incubated at 37°C and 200 rpm for 3 h. The number of CFU/mL was determined prior and at the end of the antibiotic treatment. Since there were no statistically significant differences in both control experiments (i.e. addition of equivalent volume of ethanol or sterile deionized water), only results for ethanol pre-treatments are shown.

### Statistical analysis

All the data presented are averages with standard deviations. Results were statistically evaluated using one-way ANOVA. Samples with *p*—values equal or lower than 0.05 were taken as statistically significant.

## Results and Discussion

**Fig 1:**
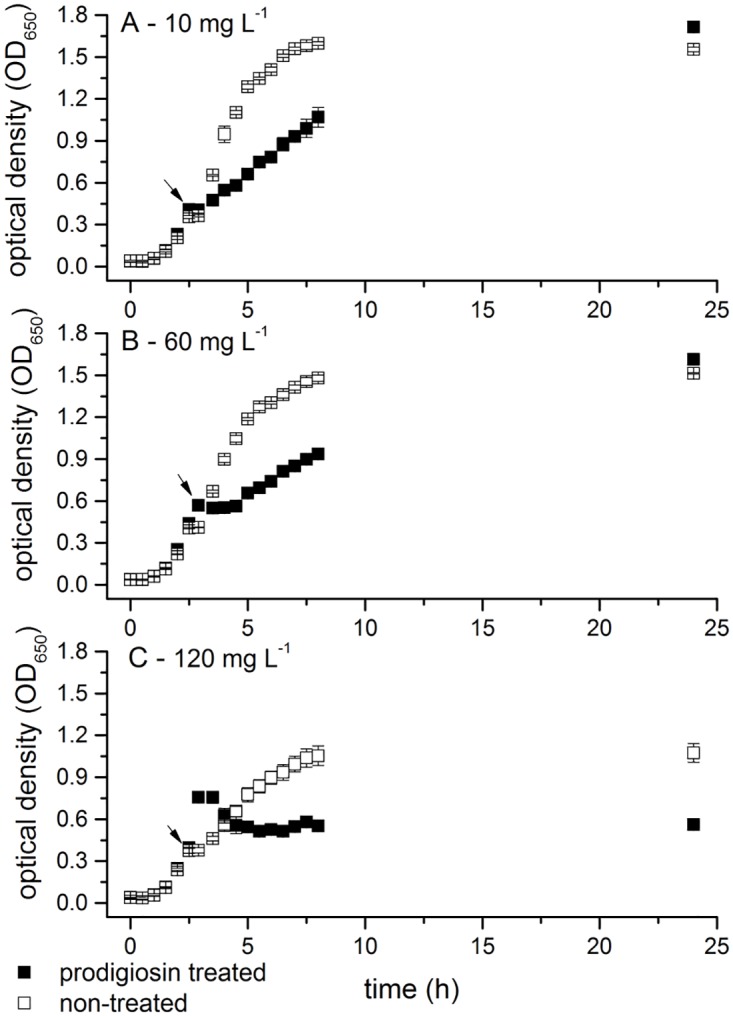
The influence of different prodigiosin concentrations on *E*. *coli* MG1655 growth. Cells were grown in LB medium at 37°C and 200 rpm and treated in the middle of the exponential phase with 10 (A), 60 (B) and 120 mg L^-1^ (C) of prodigiosin (filled symbols) or non-treated control (open symbols). The arrows represent the time of prodigiosin or ethanol addition. Data are presented as averages and standard deviations (n = 3).

Despite extensive research of prodigiosin [3,9–15], the mechanism of its antibacterial activity remains poorly understood. To address physiological response to prodigiosin, *E*. *coli* cells were grown at optimal conditions. Prodigiosin was added to the growing culture in the middle of the exponential phase. If 120 mg L^-1^ prodigiosin was added immediately after culture inoculation, no growth of *E*. *coli* was observed. As given in Fig 1 prodigiosin added at concentrations 10 and 60 mg L^-1^, which are below MIC value (103.4 mg L^-1^), reduced the growth rate of *E*. *coli* 2.5 and 3 fold, respectively. Beside this, the addition of 60 mg L^-1^ of prodigiosin induced a two-hour growth arrest (Fig 1B). Nevertheless after 24 hours of incubation optical density of control and prodigiosin treated cultures were comparable. At concentrations above MIC (i.e. 120 mg L^-1^) *E*. *coli* population ceased to grow immediately after the addition of prodigiosin (Fig 1C). The transient increase in optical density is attributed to the added prodigiosin, which was subsequently incorporated in the bacterial cells, and not to the bacterial growth. At the end of the incubation there was a significant difference in optical density between the control and prodigiosin treated cultures. The amount of prodigiosin incorporated into the *E*. *coli* cells was dependent on the applied concentration (i.e. 22.5 mg L^-1^ or 41.1 mg L^-1^, when treated with 60 mg L^-1^ or 120 mg L^-1^ of prodigiosin, respectively). This is approximately 30–40% of the added amount. Comparable amount of incorporated prodigiosin was found in inactivated autoclaved cells (46.7 ± 0.8 mg L^-1^ versus 41.0 ± 1.0 mg L^-1^ in viable cells), indicating that incorporation of prodigiosin was not coupled to an active metabolic process. Due to its physicochemical properties prodigiosin most likely accumulated into the lipid bilayer [3,34], although prodigiosin presence in the cytoplasm has been observed as well [35].

**Fig 2:**
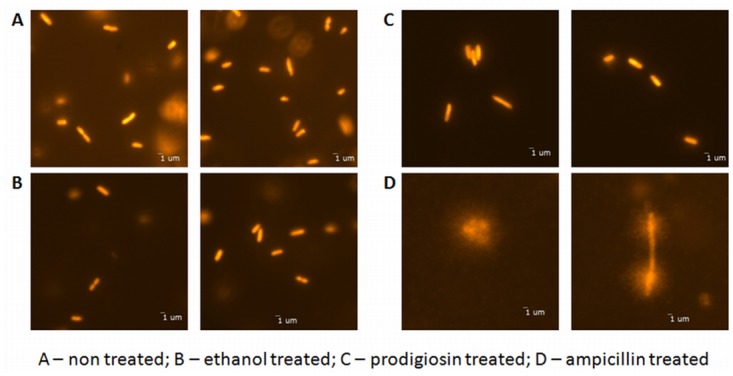
The modified comet assay for studying prodigiosin addition to *E*. *coli* MG1655 cells grown in LB medium at 37°C and 200 rpm. A—non-treated bacterial cells; B—control cells treated with 4.44% (V/V) of ethanol; C—cells treated with 120 mg L^-1^ of prodigiosin; D—positive control cells treated with 100 mg L^-1^ of ampicillin. Cells were treated in the middle of the exponential phase and were inspected 1 h after the treatment. Cells were stained with GelRed^™^ and observed by epifluorescence microscopy. The scale bar represents 1 μm.

**Fig 3:**
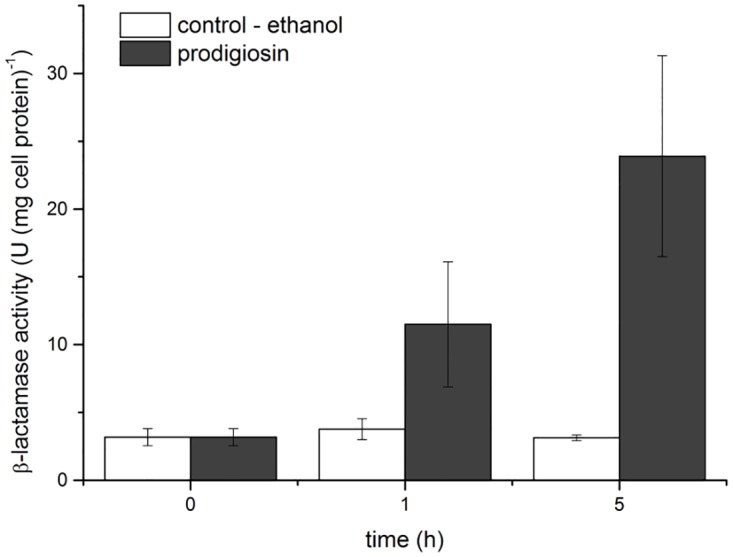
β—lactamase activity of *E*. *coli* MG1655 cells determined as nitrocefin hydrolysis. The control (white columns), cells treated with 120 mg L^-1^ prodigiosin (black columns). Cells were grown in LB medium at 37°C and 200 rpm. Data are presented as averages and standard errors (n = 3).

Modified comet assay was used to assess whether prodiogiosin compromise *E*. *coli* cell membrane and DNA integrity. In the prodigiosin treated culture majority of the cells were intact and we found only few with emanating fluorescence halo that indicates cell membrane disintegration and DNA leakage (Fig 2). No such damage was caused, when cells were treated with ethanol or were keept only in growth medium (negative control). On the other hand, when cells were treated with cell wall targeting antibiotic ampicillin (positive control), DNA leaked out of the cells and produced a visible fluorescence halo around the cells. The viability and membrane integrity have also been checked with Live/Dead BacLight viability assay (Table 1). Prodigiosin affected cell viability after one hour (p < 0.001) and approximately 15% drop in viability was determined, which did not changed with further incubation (Table 1). At the same time, the control (ethanol treated cells) remained viable for 5 hours. We conclude that prodigiosin has a minor effect on *E*. *coli* cell viability and that the majority of cells had intact membranes after prodigiosin treatment (Fig 2, Table 1). This is in sharp contrast to *Bacillus subtilis* cells were prodigiosin at much lower concentrations caused an efficient and quick cell lysis [15]. To check if the outer membrane of *E*. *coli* is compromised after prodigiosin treatment, a nitrocefin hydrolysis assay was used [29]. In case of leakage, β –lactamase is released from the cell periplasm and degrades nitrocefin, which is present in the medium. The results presented in Fig 3 show that β –lactamase activity of prodigiosin treated cells was significantly higher already after one hour of treatment with prodigiosin and increased even more after 5 hours of treatment as compared to the control. The results imply that the outer membrane, but not the cytoplasmic membrane, is damaged after prodigiosin treatment. It should be noted, however, that prodigiosin treated cells were morphologically different compared to the control cells. After 21.5 h of incubation, the control cells were 1.9 ± 0.2 μm rod-like shaped, whereas cells treated with 120 mg L^-1^ of prodigiosin were double the size, measured on average 3.6 ± 0.4 μm in length. Prodigiosin treated cells did not multiply; there were no changes in CFU counts after the treatment. This suggests that cells increase their biomass, but were not able to divide after prodigiosin treatment. Cell elongation is typical for cells in which SOS response has been induced. SOS induction is linked to significant DNA damage [36]. It is generally accepted that in eukaryotic cells prodigiosin causes DNA cleavage and fragmentation in addition to cell acidification, cell cycle arrest, activation of caspase activity, and interference with signal transduction pathways [3,13,37]. There are, however, no reports of corresponding DNA cleavage or fragmentation induced by prodigiosin in bacterial cells. To check if prodigiosin can cause DNA damage in *E*. *coli*, chromosomal DNA was isolated and treated with prodigiosin. Irrespective of the pigment concentration no DNA fragmentation was observed. In SOS induced cells SulA inhibits FtsZ ring formation and prevents cell division leading to cell elongation [38]. To check if SOS was indeed induced with prodigiosin treatment, a reporter gene *gfp* fused to the promoter of *sulA* gene was used. This allowed us to monitor the induction of *sulA* transcription and therefore the activity of the SOS response *in vivo* [31,39]. UVC exposed cells induced *sulA* transcription, on the other hand, no transcription was detected in prodigiosin treated cells suggesting that SOS response was not induced upon prodigiosin treatment in *E*. *coli* cells.

**Table 1:** Viability of *E*. *coli* MG1655 cells treated with prodigiosin. Cells were grown in LB medium at 37°C, 200 rpm, and inspected under inverted epifluorescence microscope before, 1, and 5 hours after addition of either 120 mg L^-1^ of prodigiosin or 4.44% (V/V) of ethanol in control samples in the middle of the exponential phase. Images were obtained using fluorescence filters for green fluorescent dye SYTO 9 and red fluorescent dye propidium iodide. Data are presented as averages and standard deviations (n ≥ 3).

Time of treatment (h)	The fraction of viable cells ethanol treated	The fraction of viable cells prodigiosin treated
**0**	0.95 ± 0.002	0.95 ± 0.002
**1**	0.96 ± 0.01	0.81 ± 0.07
**5**	0.96 ± 0.01	0.8 ± 0.01

**Fig 4:**
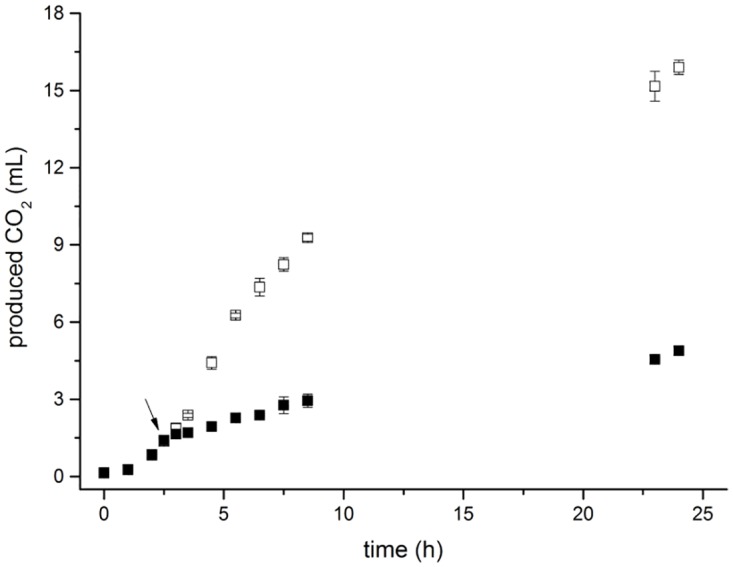
CO_2_ production of *E*. *coli* MG1655 cells treated with prodigiosin. Cells were treated with 120 mg L^-1^ of prodigiosin (filled symbols) during incubation in LB medium at 37°C and 200 rpm, control—ethanol treated (open symbols). CO_2_ was measured by gas chromatography. The arrow represents the time of prodigiosin or ethanol addition. Data are presented as averages and standard deviations (n = 4).

Total bacterial metabolic activity of prodigiosin treated *E*. *coli* cells was measured by respiration (Fig 4). Control bacterial culture showed a typical growth-dependent pattern of CO_2_ production, whereas cells treated with 120 mg L^-1^ of prodigiosin exhibited severely impaired metabolic activity. Prodigiosin inhibited CO_2_ production by four-fold at the end of incubation, indicating interference with citric acid cycle and pentose phosphate pathway. As a consequence, prodigiosin treated *E*. *coli* cells may have generated fewer ATP molecules. It has been proposed that prodigiosin inhibits F-ATPases in *E*. *coli in vitro* [40]. Lower cell energy charge influences transcription, translation [41] as well as cell division [42]. The effect of prodigiosin treatment on total RNA and protein content is given in Table 2. The total protein content in prodigiosin treated cells did not change (p-value 0.77) with incubation. This is in agreement with the absence of net growth and optical density results and suggests that prodigiosin may interfere with *de novo* protein synthesis in *E*. *coli*. In a control there was a significant two-fold increase in total protein content (p = 0.5 · 10^−4^ for ethanol treated, and p = 1 · 10^−4^ for deionized water treated cells) after two hours of incubation. Similar results were obtained for the total RNA content of *E*. *coli* cells (Table 2). No increase in the total RNA level after prodigiosin treatment implies that prodigiosin may interfere with the transcription process. This does not necessarily contradict with the finding that cell size has increased after prodigiosin treatment. The existing complement of proteins and RNA could still be synthetically active. Presently, it is not known if prodigiosin interferes directly or indirectly with the process of transcription.

**Table 2:** Protein and total RNA content in *E*. *coli* MG1655 cells treated with prodigiosin. Cells were treated with 120 mg L^-1^ prodigiosin, 4.44% (V/V) ethanol or sterile deionized water as a control in the middle of the exponential phase. Data are presented as averages and standard deviations (n ≥ 3).

		Protein content (mg protein cell^-1^)	Total RNA content (ng μL^-1^)
**before treatment**		(2 ± 0.5) · 10^−11^	469 ± 25
**2 h after treatment**	**prodigiosin**	(1.9 ± 0.5) · 10^−11^	384 ± 22
	**ethanol**	(4.2 ± 0.7) · 10^−11^	521 ± 5
	**deionized water**	(3.7 ± 0.5) · 10^−11^	652 ± 49

The results suggest that prodigiosin has a bacteriostatic effect on *E*. *coli* cells above its MIC value. Prodigiosin does not kill or lyse *E*. *coli* cells, but only inhibits their division and metabolic activity. After re-inoculation of prodigiosin arrested cells into a fresh growth medium that did not contain prodigiosin, growth was resumed. When cells were subsequently treated with prodigiosin, transferred to fresh medium and treated with prodigiosin, they consistently stop multiplying after each prodigiosin treatment, but recovered after re-inoculation into the fresh prodigiosin-free medium. This clearly suggests that prodigiosin is a bacteriostatic agent for *E*. *coli*. The Malthusian fitness of treated cells was between -0.6 and 0.25 and did not differ significantly between consequtive prodigiosin treatments, indicating that *E*. *coli* did not become resistant to prodigiosin.

**Fig 5:**
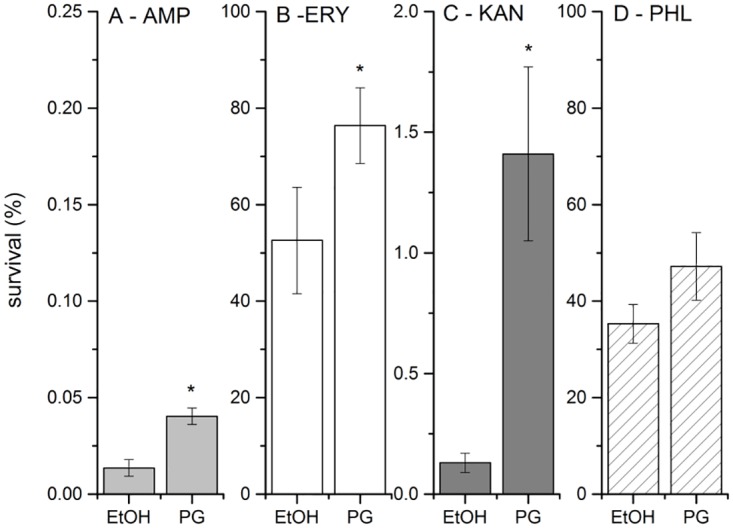
Survival of *E*. *coli* MG1655 cells pre-treated with prodigiosin followed by treatment with antibiotics. Cells were pre-treated with 120 mg L^-1^ prodigiosin (PG) or in control with ethanol (EtOH). Tested antibiotic concentrations were 100 mg L^-1^ ampicillin (A), 100 mg L^-1^ erythromycin (B), 100 mg L^-1^ kanamycin (C) and 5 mg L^-1^ phleomycin (D). Data are presented as averages and standard deviations (n = 3). Asterisks represent statistically significant differences between ethanol and prodigiosin pre-treatment (p < 0.05). Note the difference in y-axis ranges.

It is known that a combination of several stress factors aggravate bacterial fitness. For example, combination of antimicrobial agents due to synergistic effect is often applied in microbial growth inhibition [43]. To check how prodigiosin in a combination with several known antibiotics affects survival of bacteria, an assay described by Dӧrr *et al*. [33] has been used. As prodigiosin interferes with protein and nucleic acid synthesis, as well as it has an effect on the outer cell membrane leakage, four antibiotics were used with different targets: erythromycin and kanamycin as inhibitors of protein synthesis, ampicillin as inhibitor of cell wall synthesis, and phleomycin as DNA damaging agent. As given in Fig 5, pre-treatment with prodigiosin significantly affected survival during the antibiotic treatment. Ampicillin affected the viability of *E*. *coli* to the highest degree and merly 0.01% survival rate was detected, when cells were treated with ampicillin alone. Prodigiosin pre-treatment surprisingly significantly increased the survival rate approximately 3-fold (p = 0.002) (Fig 5A). When cells were exposed to erythromycin, the survival rate was approximately 50% (Fig 5B), but increased significantly to 75% (p-value 0.04), if cells were pre-treated with prodigiosin prior to erythromycin addition. A similar effect was observed in kanamycin treatment, also an inhibitor of protein synthesis. Only approximately 0.15% of cells survived the kanamycin treatment (Fig 5C), however, an approximately 10-fold greater survival was observed in prodigiosin pre-treated cells (p-value 0.006). The obtained results are surprising and indicate that prodigiosin interferes with selected antibiotics, which inhibit protein and cell wall synthesis. Since prodigiosin may adsorb to proteins [44], it is feasible that prodigiosin could be present on ribosomes, where it may interfere with the action of ribosomal antibiotics. On the other hand, prodigiosin intercalates in the membrane and may interfere with antibiotics that inhibit cell wall biosynthesis. As antibiotics need active cells for the maximum effect, the shut down of cell activity by prodigiosin pre-treatment may have inadvertently protected cells from the action of antibiotics. In contrast, prodigiosin pre-treatment had no significant effect (p-value 0.06) on the survival of *E*. *coli* cells treated with phleomycin (Fig 5D). This is consistent with the results of the induction of SOS response and DNA fragmentation and indicates that DNA is not the main target for prodigiosin in *E*. *coli*.

The results of antibacterial activity of prodigiosin, obtained in this study, imply that prodigiosin impedes several key metabolic functions in the *E*. *coli* cell (i.e. RNA and protein synthesis, cell division, outer membrane integrity, cell respiration). In this respect, it has a pleiotropic effect on *E*. *coli* cell metabolism. For a small molecule that is foreign to the organisms such as prodigiosin and is not actively metabolised in the cell, it is not unusual to have interactions with several different molecules [45]. Due to its physico-chemical nature, prodigiosin may be found in different cell compartments and interfere non-specifically with diverse cellular processes. As microbiologists we are all too often focused on very specific interactions based on proteins that require high fidelity in order to proceed. However, from a bacterial point of view a potent antimicrobial molecule could either be very specific or broad spectrum low toxic molecule that obstructs several cellular processes, thereby reducing the overall fitness of the adversary. Interestingly, depending on the context, prodigiosin appears to posses both features. As we have shown in our previous paper, prodigiosin in low concentrations specifically activates autolysins that in less than an hour after application completely auto-destroy *Bacillus subtilis* [15]. The same molecule, however, has a completely different mode of action in *E*. *coli* cells, where it attacks several cell targets, arrest overall growth, but enables the cell an escape if diluted. In this respect, prodigiosin epitomize all the beauty and complexity of small molecules interacting with the biological macromolecules to which they can diffuse to and interact with. The biology of small molecules is in the age of molecular biology, which almost exclusively focuses on macromolecules, seriously underrepresented. It is for this reason that classical physiological studies have merit in microbiology and should be fostered as we now have tools to elucidate the underlying mechanisms.

## Conclusions

The results presented in this work demonstrate that prodigiosin acts as a bacteriostatic agent on *E*. *coli* and influences several physiological processes or behaviours. Cytoplasmic membranes and chromosomal DNA remained largely unaffected by prodigiosin treatment. The outer membrane becomes leaky. Growth, cell division, protein and RNA synthesis, as well as the overall metabolic activity, were severely impaired after prodigiosin treatment in particular above MIC values. Prodigiosin´s bacteriostatic activity can be reverted if prodigiosin is diluted when *E*. *coli* cells resume growth. Unexpectedly, pre-treatment with prodigiosin improves survival of antibiotic treatment.
